# Surface Response of Brominated Carbon Media on Laser and Thermal Excitation: Optical and Thermal Analysis Study

**DOI:** 10.1186/s11671-017-1873-7

**Published:** 2017-02-23

**Authors:** Volodymyr V. Multian, Fillip E. Kinzerskyi, Anna V. Vakaliuk, Liudmyla M. Grishchenko, Vitaliy E. Diyuk, Olga Yu. Boldyrieva, Vadim O. Kozhanov, Oleksandr V. Mischanchuk, Vladyslav V. Lisnyak, Volodymyr Ya. Gayvoronsky

**Affiliations:** 1 Institute of Physics, National Academy of Science of Ukraine, Prospect Nauky 46, Kyiv, 03680 Ukraine; 2 Taras Shevchenko National University of Kyiv, Chemical faculty, Volodymyrska Str. 62a, Kyiv, 01601 Ukraine; 3 O.O. Chuiko Institute of Surface Chemistry, National Academy of Science of Ukraine, General Naumov Str. 17, Kyiv, 03164 Ukraine

**Keywords:** Elastic optical scattering, Porosity, Bromine, Thermal desorption, Carbon textile

## Abstract

The present study is objected to develop an analytical remote optical diagnostics of the functionalized carbons surface. Carbon composites with up to 1 mmol g^−1^ of irreversibly adsorbed bromine were produced by the room temperature plasma treatment of an activated carbon fabric (ACF) derived from polyacrylonitrile textile. The brominated ACF (BrACF) was studied by elastic optical scattering indicatrix analysis at wavelength 532 nm. The obtained data were interpreted within results of the thermogravimetric analysis, X-ray photoelectron spectroscopy and temperature programmed desorption mass spectrometry. The bromination dramatically reduces the microporosity producing practically non-porous material, while the incorporated into the micropores bromine induces the dielectric and structural impact on surface polarizability and conductivity due to the charging effect. We have found that the elastic optical scattering in proper solid angles in the forward and the backward hemispheres is sensitive to the kind of the bromine bonding, e.g., physical adsorption or chemisorption, and the bromination level, respectively, that can be utilized for the express remote fabrication control of the nanoscale carbons with given interfaces.

**Electronic supplementary material:**

The online version of this article (doi:10.1186/s11671-017-1873-7) contains supplementary material, which is available to authorized users.

## Background

Nanoscale graphenes and related nanographite network systems have open edges, which adopt a nonbonding *π*-electron state [1–3]. This edge state exists at the degenerate point between the graphitic *π* and π* bands [4] and, definitely, it plays a role of the electron reservoir. As a result, the state population dynamic can effect on electronic/magnetic and optical, including nonlinear optical, properties [5–7]. A random disordered 3D structure of activated carbon fibers (ACFs) is frequently considered as a flexible network of the nanographite domains. Each domain originates from stacked graphene planes [3, 8] within interstitial nanosized voids—micropores [9]. The intercalation and guest-host interactions [2, 8, 9], including molecular adsorption, affect the properties of ACFs through the downshift of the Fermi level [2, 4, 5, 10]. Previously in [2], the bromination impact on the nanographite network magnetic properties was examined. Among the different kinds of nanographite, microporous ACFs provide an important model system with a variety of specific magnetic features manifestation related to its unique electronic structure [3, 10, 11]. The ACFs materials demonstrate an advanced response to the inclusion. The molecules and ions accommodated into the voids induce the charging effect, spin glass states [11–14], and can cause the magnetic switching [15, 16] and edge-state gas sensing [3, 8–12, 17]. So, the controlled bromination can be involved in the production of carbon interfaces for nanoscale sensing, charge/spin current modulation, nonvolatile magnetic switching, and optoelectronic applications [3, 11–13, 18–24]. The yield of bromine grafted over carbons by adsorption might be insufficient, while the radio frequency plasma bromination with the treatment time control is the reasonable route for the inclusion of the preset Br quantity. The plasma bromination gives much higher bromine content, up to 1.0 mmol g^−1^ of Br. In contrast to the high-temperature gas-phase halogenation [25], the high content of target surface functionalities could be reached even at the room temperatures. This extra quantity of the grafted bromine can be utilized for substitution and functionalization of various graphite carbons [18] with organic molecules to be implemented in nanoscale devices [21–24].

In order to monitor surface chemistry changes during the treatment process, express diagnostics methods should be elaborated for readout bromine guest-host interaction at the nanographite interface. The obtained data can be crucially important for the precise control of the carbons interfaces fabrication for specific applications. For this purpose, we applied elastic optical scattering indicatrix analysis. Comparison of optical scattering characterization with data of X-ray photoelectron spectroscopy (XPS) and temperature programmed desorption mass spectrometry (TPD-MS) techniques have shown the promising result for diagnostics application.

## Methods

**Fig. 1:**
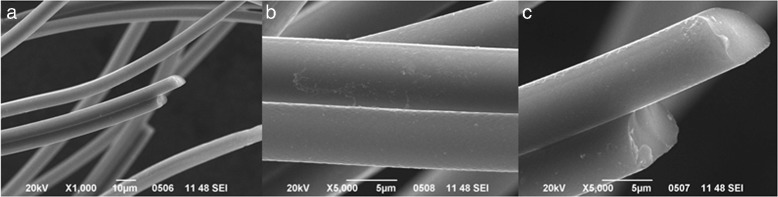
SEM micrographs. **a**, **b** initial ACF fibers, and **c** bromine treated BrACF60

In this study, we examined a representative of the flexible microporous materials class – activated carbon fabric (ACF) that prepared from polyacrylonitrile textile by carbonization and steam activation. Nitrogen adsorption-desorption was measured on a TriStar Micromeritics C10900A porosimeter at −196 °C. The ACF has Brunauer-Emmett-Teller (BET) surface area *S*_BET_ of ∼900 m^2^ g^−1^ and the total pore volume *V*_tot_ of 0.19 cm^3^ g^−1^. According to scanning electron microscopy data-electron dispersion X-ray analysis (SEM-EDX) the ACF contains C = 92.7 mass% and O = 6.43–6.55 mass%. A cylindrical plasma treatment system for the radio frequency glow discharge fed with bromine (5ml min^−1^) was used for the surface functionalization. The (27.12 MHz) plasma treatment time was from 5 to 100 min at low pressure, ∼2 Pa. Hereafter, the brominated samples were assigned as BrACF “number”, where the “number” corresponds to the treatment duration in minutes. After the plasma treatment, SEM imagery on a Jeol JSM 7700 F microscope shows a practically flat surface, without new erosions and cracks that are observed at micrometer scale level (see Fig. 1).

Surface chemistry characteristics of the brominated ACF were studied by means of TPD-MS. Continuous scanning for the selected m/z ratio (positive mode) was done versus the temperature; at the residual pressure of ca. 10^−4^ Pa. TPD-MS data were registered, at a heating rate of 10 ° C min^−1^, according to the conditions described in [25, 26] and approved in [27]. XPS analysis was performed with a JPS-9030 photoelectron spectrometer at AlK *α*, 1486 eV.

**Fig. 2:**
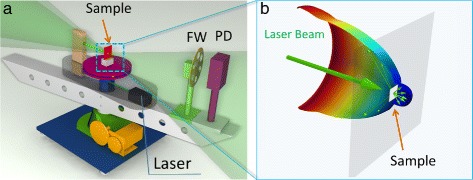
**a** Experimental setup for the cross-section of the elastic optical scattering indicatrix measurements. *FW* neutral filters wheel, *PD* photodiode. **b** 3D reconstruction of the optical scattering indicatrices in forward and backward hemispheres

Cross-sections of the elastic optical scattering indicatrix in forward and backward hemispheres were studied at the designed experimental platform (Fig. 2 a, b). A TEM_00_ beam of the CW DPSS laser at wavelength 532 nm, power *P* = 20 mW, was used as a light source. The samples were positioned perpendicularly to the incident beam and centered at the rotation axis of the setup. A diameter of the laser spot at the sample was about 3 mm that provides proper spatial averaging from the layers of the overlapped carbon fibers. Angular scattered light distribution measurement *P*(*θ*) was performed with a silicon photodiode PD (⌀ = 1 cm) placed on the rotation arm at the distance of 39 cm that corresponds to a solid angle ∼0.5 msr. The signal readout was performed by a 12-bit acquisition card with the sampling rate of 1000 samples per second. The setup acquisition dynamic range was extended up to eight orders of magnitude with neutral optical filter wheel FW insertion in front of the PD. A stepper motor with a self-locking worm gearbox provides full range (360°) rotation of the PD unit with an angular resolution of ∼ 0.05°. Overlapping of the PD unit with the incident laser beam during the angular scan produces a “blind” sector for the data acquisition that is presented as a hatched area in Fig. 4 a.

## Results and Discussion

From the Volhard titration [25] and the EDX analysis of the BrACFs, the total bromine content, *C*_Br_, is within 0.11–0.99 mmol g ^−1^ (see Additional file 1: Figure S1). It was shown that the treatment duration non-monotonously effects on *C*_Br_, it reaches the maximum for BrACF60. Prolongation of the treatment—from 60 to 100 min—reduces the *C*_Br_, due to the low-temperature discharge impact on the grafted bromine (Table 1).

**Table 1:** Thermal desorption properties of brominated ACF. Analysis of TPD-MS profiles at m/z 79 and 81

Sample	*C*_Br_	*β*_1_(Br)		*β*_2_(Br)		*β*_3_(Br)		*β*_4_(Br)		Br _(1+2)_	Br _(3+4)_	*I*_Br_
		%1 / Br _(1)_	*T*_(1)_	%2 / Br _(2)_	*T*_(2)_	%3 / Br _(3)_	*T*_(3)_	%4 / Br _(4)_	*T*_(4)_			
BrACF5	0.11	20.7 / 0.72	164	25.7 / 0.89	241	38.6 / 1.34	309	15.0 / 0.52	535	1.61	1.86	31.5
BrACF10	0.21	8.7 / 0.23	145	0.8 / 0.02	228	35.8 / 0.93	373	54.7 / 1.43	564	0.25	2.36	12.4
BrACF15	0.58	19.6 / 2.10	161	20.0 / 2.14	243	31.8 / 3.39	365	28.5 / 3.05	547	4.24	6.44	18.4
BrACF30	0.46	14.3 / 1.58	183	41.2 / 4.54	273	18.0 / 1.98	425	26.4 / 2.90	554	6.11	4.88	23.9
BrACF60	0.99	1.6 / 0.19	195	3.7 / 0.43	317	53.8 / 6.23	418	40.9 / 4.74	547	0.61	10.97	11.7
BrACF100	0.59	0.3 / 0.02	184	0.0 / 0.0	-	13.6 / 0.88	372	86.0 / 5.53	496	0.02	6.41	10.9

The total bromine concentration (*C*_Br_, mmol g ^−1^), the content (Br _(*#*)_, 10 ^−5^ mol g ^−1^), the ratio (*%* *#*, mol.%), and Br ^+^ desorption peak temperature (*T*_(*#*)_,°C) for *β*_*#*_(Br) form; the fraction of Br ^+^ (m/z 79 and 81) ion current in TPD-MS analysis (*I*_Br_, %). The *I*_Br_ was calculated as the integral intensity with account of HBr ^+^. The content Br _(*#*)_ determined by multiplying %#, *I*_Br_ and *C*_Br_

From the BET adsorption measurements, the bromination significantly decreases the microporosity resulting in an almost non-porous material production. The *S*_BET_ value reduces from 900 to 3–10 m^2^ g ^−1^. The bromine accommodated into the micropores induces the dielectric and structural effects, including a polarizability of the interface layer modification [3]. In certain bromine substituents, p electrons are conjugated with *π* electrons in an aromatic ring [28, 29]. Moreover, a bromine substituent is a weak electron-withdrawing group so that *π* electrons would be delocalized [30]. The light absorption range of a compound containing a bromine substituent is broader than that for the initial one and should exhibit a redshift. Therefore, the *ε* value of the bromine-containing compound is higher than that of the corresponding reference compound. These variations can be readout by the elastic optical scattering analysis [31]. The SEM and N_2_ adsorption data testify that the bromination reaction takes place at the ACFs surface leading to sites formation enriched with grafted bromine—a kind of the nanoscale islands on the substrate interface. The sites are relatively hydrophobic due to the bromine terminal group. An interaction potential among the sites and its decay scale can be described in terms of the interaction of Br with the carbon interface states. This interaction is characterized by recording the Br 3d core level with an assistance of XPS.

According to the XPS data, the Br 3d peak shifts to a higher binding energy after ACF Br_2_ plasma treatment (see spectra 1 and 2 in Additional file 1: Figure S2). It indicates a Br _2_ chemical state modification due to the bromine interaction with surface giving, being resulted in C _*n*_–Br_2_ nanoscaled islands coverage of the fibers interface. The reference data [32, 33] attributed Br 3d _5/2_ peak at 67.4 ±0.2 eV to the strongly bonded Br in C _*n*_–Br_2_ interface complexes, while the peak at 69.2 ±0.2 eV – to the physisorbed Br or surface-bonded HBr. The Br 3d peak at 71.0 ±0.2 eV corresponds to the bromine covalent bonded to sp^2^ and sp^3^ hybridized C atoms [34–36]. Taking into account 1.05 eV splitting between 3d _5/2_ and 3d _3/2_, the Br 3d core level spectra can be attributed to the formation of all of the mentioned forms (see Additional file 1: Figure S2). The BrACF60 spectrum reflects the dominant contribution manifestation of the Br peak of the C–Br bond at ∼71.0 eV [36] with minor contributions of the contaminant species at 68.5 eV for Br ^−^ and Br_2_ at 67.4 eV [35]. The contaminant species contribution into the spectrum is negligible for the BrACF100. A tiny dispersion of the chemical shifts of the Br-species grafted on the surface significantly limits the application of XPS for determining an origin of the response.

**Fig. 3:**
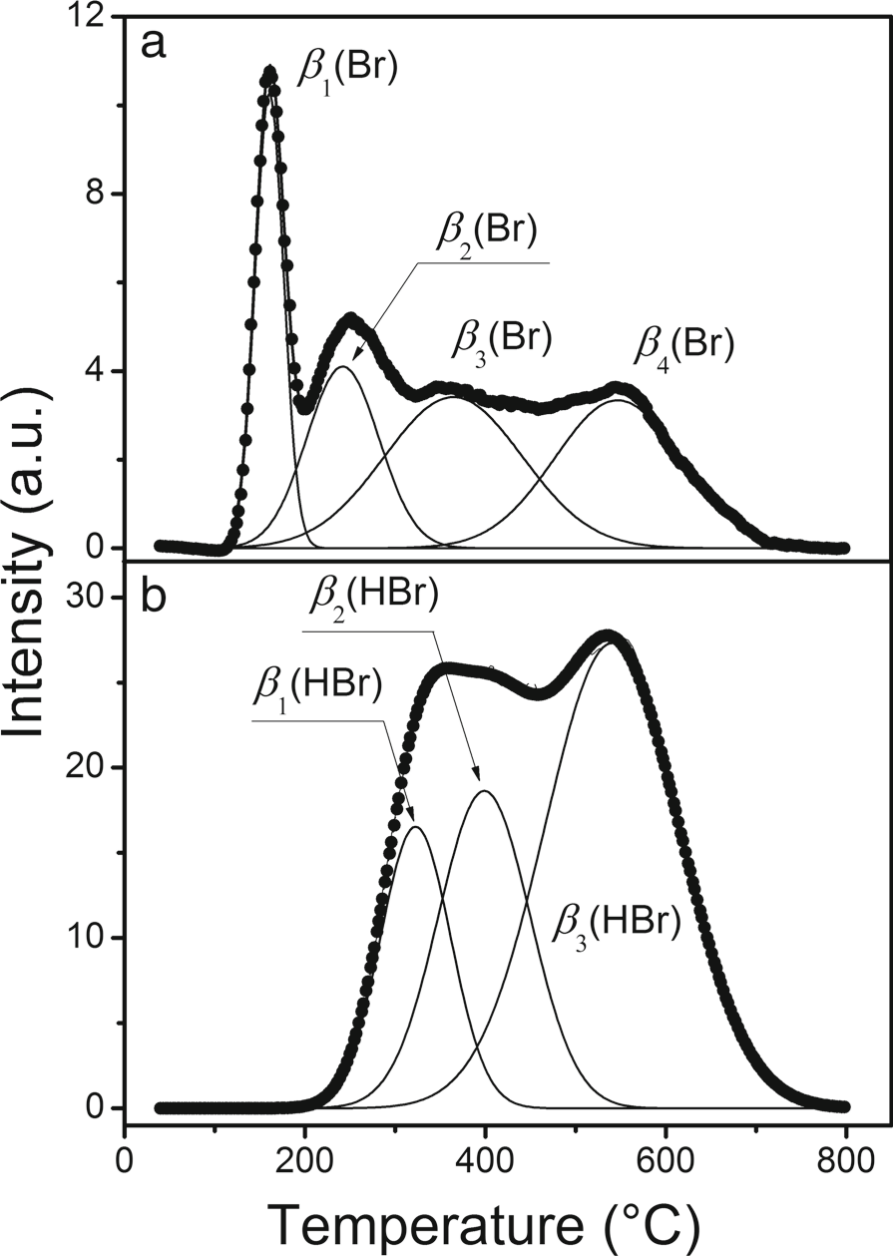
Deconvolution of the TPD-MS profiles corresponding to the m/z 79 **a** and 80 **b** for BrACF15

Chemical derivatization was performed within the thermal excitation application. According to the TPD-MS data, the surface bromine desorbs from the ACFs surface via positive ionic fragments of m/z 79 and 81 (Br ^+^) and 80 and 82 (HBr ^+^). Typical temperature profiles of Br ^+^ and HBr ^+^ are presented in Fig. 3 a, b (see Additional file 1: Figure S3 for the rest ones). The profile data integration gives the following estimations of the integral intensity of HBr (*I*_HBr_) and Br (*I*_Br_) in the bromine-containing desorption products: 68.5–89.1 and 10.9–31.5% correspondingly. Consequently, the main gaseous product of surface grafted Br thermolysis is HBr. The molecular HBr desorbs at 250–700 °C.

The Br ^+^ profiles (Fig. 3 a, Additional file 1: Figure S3a-e) can be deconvoluted into a set of four components. Each component can be separated due to the pronounced difference of the desorption peak temperatures, see T _(1−4)_ in Table 1. The desorption bands of different Br ^+^ species were registered in the temperature range of 145–195 °C (*β*_1_(Br)), 228–317 °C (*β*_2_(Br)), 309–425 °C (*β*_3_(Br)), and 496–564 °C (*β*_4_(Br)). These components can be attributed to the distinct surface centers adopting the bromine species. The centers have comparable bond strength with a certain type of bromine. They have also small differences of the thermal destruction and HBr desorption energies.

The HBr ^+^ profiles (Fig. 3 b, Additional file 1: Figure S3a*-e*) were deconvolved into three component set with HBr ^+^ desorption band of 304–385 °C (*β*_1_(HBr)), 397–476 °C (*β*_2_(HBr)), 541–583 °C (*β*_3_(HBr)) that are shifted towards the ranges of the *β*_*#*_(Br) set. The profiles show a certain distribution of Br-bounded centers that differentiate by the desorption energy (Additional file 1: Figure S3a*-e*). So, it is difficult to draw a clear temperature boundary between *β*_*#*_(Br) components.

We attributed *β*_1_(Br), *β*_2_(Br), *β*_3_(Br), and *β*_4_(Br) components to the following bromine forms: (1) physisorbed, (2) physisorbed or intercalated in micropores, (3) chemisorbed on a more accessible surface, and (4) chemisorbed in tight micropores, correspondingly.

The less intense and fully symbate profiles of m/z 79 (Br ^+^) and 80 (HBr ^+^) at the temperature >300 °C can be explained by the HBr ^+^ dissociation in an ionic trap of the mass spectrometer. At the low-temperature mode < 300 °C the profiles of desorption of Br ^+^ and HBr ^+^ do not coincide due to the low-temperature desorption of physisorbed *β*_1_(Br) form and a fraction of weakly-bonded bromine *β*_2_(Br) forms.

A prolongation of the ACF plasma treatment induced (i) reducing the fraction of *β*_1_(Br) and *β*_2_(Br) with an increase of the contribution of *β*_3_(Br) and *β*_4_(Br) forms; (ii) the absolute content of *β*_1_(Br), *β*_2_(Br) and *β*_3_(Br) in BrACFs passes through maxima at 15, 30, and 60 min of the treatment, respectively; (iii) the absolute content of the *β*_4_(Br) substantially increases with the treatment time, (iv) a rise of the HBr fraction in the desorption products, see for details Additional file 1: Figure S3a*-e* and Tables 1 and 2. Based on the obtained results, the plasma bromination caused the gradual transformation of the physisorbed into the chemisorbed bromine that bound the most active centers of the ACF surface. The reductive properties of carbon surface and chemisorbed H (even presented in the form of adspecies) support the formation of HBr, as the major product of the surface bromine thermodesorption. The temperature increase over 300 °C promotes the breakage of the C–Br and C–H bounds. The free species are recombined into molecular HBr that desorbs under vacuum conditions.

**Table 2:** Thermal desorption properties of brominated ACF. Analysis of TPD-MS profiles at m/z 80 and 82

Sample	*C*_Br_	*β*_1_(HBr)		*β*_2_(HBr)		*β*_3_(HBr)		*I*_HBr_
		%1 / HBr _(1)_	$T^{*}_{(1)}$	%2 / HBr _(2)_	$T^{*}_{(2)}$	%3 / HBr _(3)_	$T^{*}_{(3)}$	
BrACF5	0.11	17.4 / 1.31	304	34.8 / 2.63	397	47.8 / 3.60	558	68.5
BrACF10	0.21	14.1 / 2.60	328	23.7 / 4.37	418	62.2 / 11.44	565	87.6
BrACF15	0.58	17.8 / 8.41	323	26.1 / 12.37	399	56.1 / 26.55	541	81.6
BrACF30	0.46	5.1 / 1.78	385	22.5 / 7.88	456	72.4 / 25.35	559	76.1
BrACF60	0.99	13.4 / 11.69	348	62.7 / 54.83	455	23.9 / 20.89	583	88.3
BrACF100	0.59	17.5 / 9.22	370	52.9 / 27.82	476	29.5 / 15.53	556	89.1

The total bromine concentration (*C*_Br_, mmol g ^−1^), the content (HBr _(*#*)_, 10 ^−5^ mol g ^−1^), the ratio (%#, mol.%) and HBr ^+^ desorption peak temperature (${T}_{\left(\#\right)}^{\ast },\kern0.3em {}^{\circ}$C) for *β*_*#*_(HBr) form; the fraction of HBr ^+^ (m/z 80 and 82) ion current in TPD-MS analysis (*I*_HBr_, %). The content HBr _(*#*)_ determined by multiplying %#, *I*_HBr_ and *C*_Br_

**Fig. 4:**
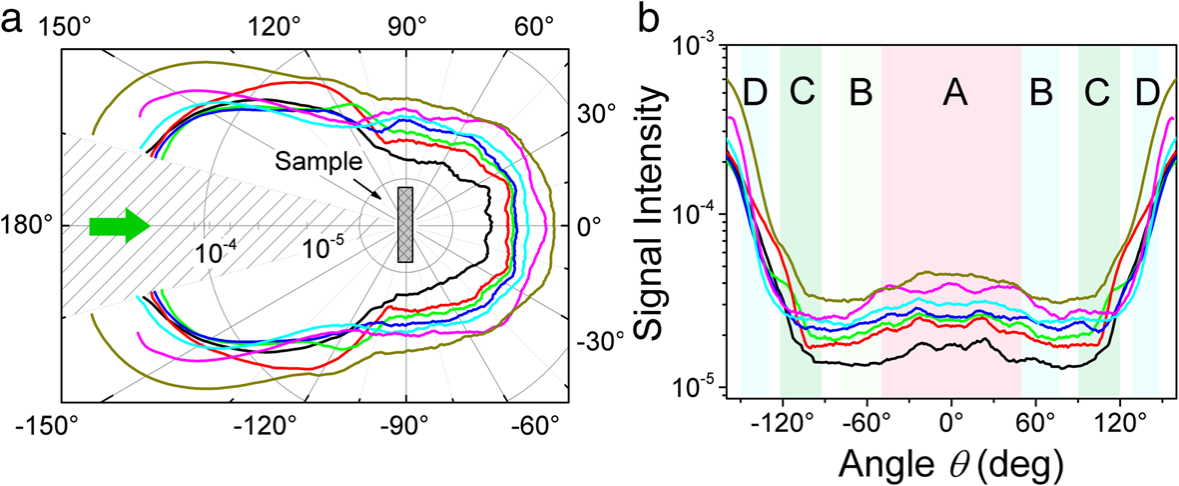
Cross-sections of the BrACFs elastic optical scattering indicatrix under CW laser excitation at 532 nm in logarithmic scale in polar (**a**) and Cartesian coordinates (**b**). *Black* ACF, *red* BrACF5, *green* BrACF10, *blue* BrACF15, *cyan* BrACF30, *magenta* BrACF60, *yellow* BrACF100

Cross-sections of the BrACFs elastic optical scattering indicatrix under CW laser excitation at 532 nm are presented in Fig. 4 a. The obtained scattering signals were normalized on the total excitation power and smoothed. The relative error did not exceed 13% for each experimental curve. In general, it is shown that the scattered signal *ε*_sc_ depends on the bromination time. Obtained polar plot can be separated into two groups of characteristic sectors (see Fig. 4 b) that correspond to the scattering in the forward (sectors **A** – |*θ*|<50° and **B** – 50°<|*θ*|<80°) and in the backward hemispheres (sectors **C** – 90°<|*θ*|<120° and **D** – 130°<|*θ*|<150°).

**Fig. 5:**
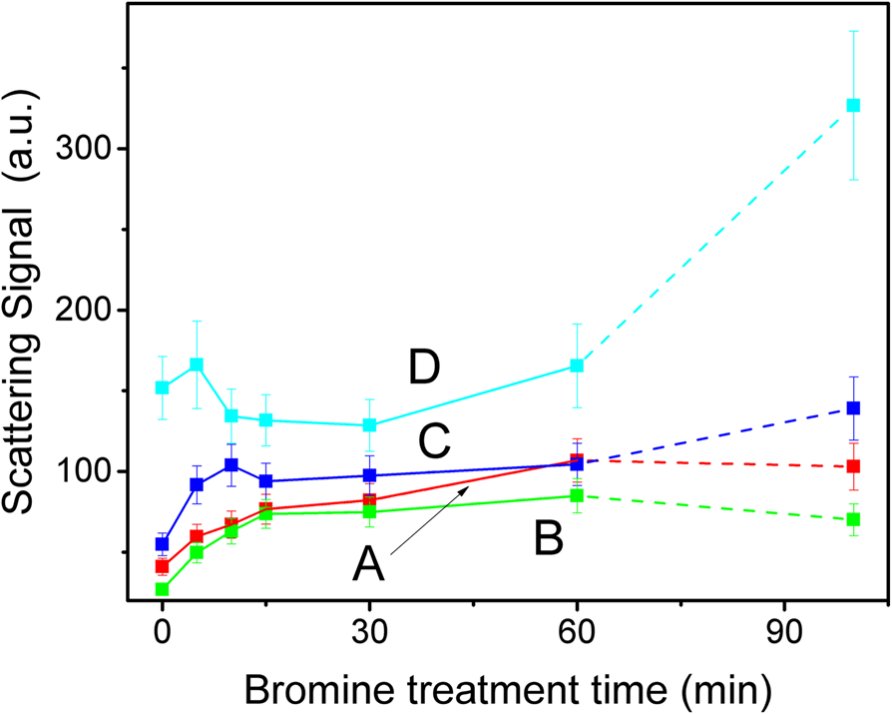
Integrated scattering signal *ε*_sc_ in cones: **A** – |*θ*|<50°, **B** – 50°<|*θ*|<80°, **C** – 90°<|*θ*|<120°, **D** – 130°<|*θ*|<150° that corresponds to the highlighted areas in Fig. 4 b

The scattering signal integrated into spherical coordinates [37, 38] for the sectors **A**, **B**, **C**, and **D** are presented in Fig. 5. The scattering in the forward hemisphere (sectors **A** and **B**) shows the monotonic growth with the bromine treatment time. In the case of scattering in the backward hemisphere (sectors **C** and **D**), the scattering magnitudes are higher and the signal demonstrates more complex dependence: an intensive growth of the signal for the short-time bromine treatment saturates with local maxima and turns to a slight monotonic growth.

**Fig. 6:**
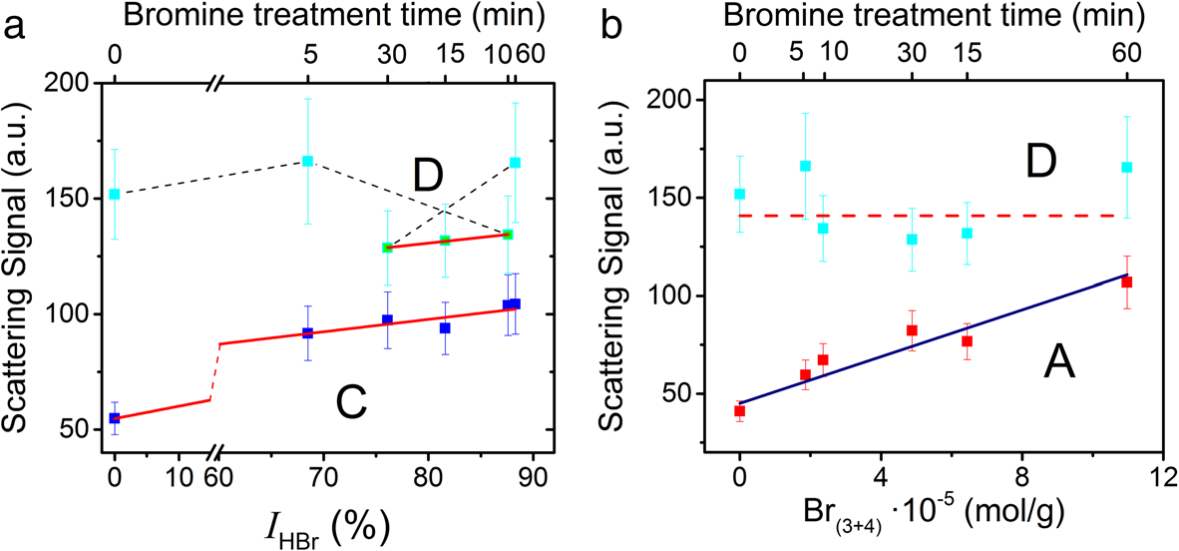
The integrated scattering signal versus **a** integral intensity *I*_HBr_ and **b** chemisorbed bromine concentration Br _(3+4)_

In order to associate an obtained experimental data with chemical content, the simple regressive analysis can be used. Each integrated curve was linearly approximated versus the concentration of the different kinds of the bromine species. The most representative results of the proposed analysis are presented in Fig. 6. The sample BrACF100 was excluded from the analyzed datasets because it shows a more complicated scattering pattern. It requires more precise study for its response explanation.

In general, the obtained results show a correlation between HBr content and the elastic optical scattering in the backward hemisphere for the large scattering angles (see sector **C** in Fig. 6 a). For the smaller backscattering angles (sector **D**), the obtained experimental dependence *ε*_sc_(*I*_HBr_) is more complex. In sector **C**, the scattering signal shows a linear dependence *ε*_sc_∝*I*_HBr_ with the factor of quality *R* ^2^=99%. In sector **D**, the dependence *ε*_sc_ on *I*_HBr_ is non-monotonous, but in the bromine treatment time range 10–30 min it has the same slope as the *ε*_sc_(*I*_HBr_) in sector **C**. The obtained non-monotonous character for sector **D** can be attributed to the scattered light redistribution in the vicinity of the exact backscattering condition (“blind” zone in Fig. 4 a). This phenomenon can be explained by the bromine incorporation into the micropores and the domination of the direct “metal” reflection from the composite surface. The last suggestion requires further research.

For the forward scattering, it was shown a linear correlation between the concentration of chemisorbed bromine Br _(3+4)_ and the scattering at “smaller” (sector **A**) angle (see Fig. 6 b). For the large scattering angle (sector **B**), the level of the signals is lower and does not allow to attribute it to the certain kind of bromine contained in the BrACF.

The comparison of the light forward and the backscattering efficiencies *ε*_sc_ in smaller angles (sectors **A** and **D**) versus the chemisorbed bromine concentration Br _(3+4)_ is presented in Fig. 6 b. The level of the backscattering signal (sector **D**) is 2–3 times higher in comparison with the forward scattering one. We did not reveal distinct dependency of the *ε*_sc_ in sector **D** on the Br _(3+4)_ magnitude. It should be noted that the total bromine concentration is proportional to the chemisorbed bromine concentration, *C*_Br_∝Br_(3+4)_. In sector **A**, the scattering signal shows a linear dependence *ε*_sc_∝Br_(3+4)_ with the factor of quality *R* ^2^=89%. The obtained result is promising for the nondestructive express analysis of the chemisorbed bromine concentration in the synthesized BrACF samples.

## Conclusions

In the present study, ACFs were brominated with a radio frequency vacuum technique. The surface functionalization was monitored in order to determine the optimal conditions for the BrACFs production. It can be used as precursors for the consequent applications. A different plasma treatment time was approved. The results indicated a possibility to functionalize ACFs with up to 1.0 mmol g ^−1^ bromine within the single step production process. From SEM imagery, the plasma bromination has a minor effect on an anterior view of the individual fibers. XPS and TPD-MS studies reveal that the interaction between ACFs and the adsorbed bromine passes mainly via covalent bonding and physisorption interactions. The physisorbed bromine accommodated into the nanopores induces the dielectric and structural impact on the surface polarizability and conductivity due to the charging effect. The obtained results of the elastic optical scattering indicatrices analysis demonstrate (i) the correlation between the scattering in the forward hemisphere and chemisorbed bromine concentration and (ii) the correlation between the scattering in the backward hemisphere and the integral intensity of HBr ^+^ in TPD-MS profiles. The proposed optical diagnostics is a promising analytical technique for the surface characterization that can be adapted to related tasks. The scattering analysis can be used for ACFs surface characterization and measurements of the physisorbed and chemisorbed bromine concentration in the carbon materials.

## Additional file

Additional file 1 Electronic Supplementary Material. (PDF 495 kb)

## Abbreviations

ACF: Activated carbon fabric
BrACF: Plasma brominated ACF
SEM: Scanning electron microscopy
XPS: X-ray
Photoelectron spectroscopy; TPD-MS: Thermoprogrammed desorption mass spectrometry
EDX: Electron dispersion X-ray
*S*_BET_: Brunauer-Emmett-Teller (BET) surface area
*V*_tot_: The total pore volume

## References

[1] Jang BZ, Zhamu A (2008). Processing of nanographene platelets (NGPs) and NGP nanocomposites: a review. J Mater Sci.

[2] Yang Z, Gao R, Hu N (2012). The prospective two-dimensional graphene nanosheets: preparation, functionalization and applications. Nano-Micro Lett.

[3] Takai K, Kumagai H, Sato H, Enoki T (2006). Bromine-adsorption-induced change in the electronic and magnetic properties of nanographite network systems. Phys Rev B.

[4] Harigaya K (2002). Theory on the mechanisms of novel magnetism in stacked nanographite. Mol Cryst Liq Cryst.

[5] Acik M, Chabal YJ (2011). Nature of graphene edges: a review. Jpn J Appl Phys.

[6] Enoki T, Ando T (2014). Physics and chemistry of graphene: graphene to nanographene.

[7] Uklein AV, Diyuk VE, Grishchenko LM, Kozhanov VO, Boldyrieva OYu, Lisnyak VV, Multian VV, Gayvoronsky VYa (2016). Characterization of oxidized carbon materials with photoinduced absorption response. Appl. Phys. B.

[8] Enoki T (2012). Nanographene-based host-guest systems. J Phys Chem Solids.

[9] Sato H (2002). Drastic effect of water-adsorption on the magnetism of nanomagnets. Solid State Commun.

[10] Enoki T, Suzuki M, Endo M (2003). Graphite intercalation compounds and applications.

[11] Enoki T, Kobayashia Y (2005). Magnetic nanographite: an approach to molecular magnetism. J Mater Chem.

[12] Kheirabadi N, Shafiekhani A, Fathipour M (2014). Review on graphene spintronic, new land for discovery. Superlattices Microstruct.

[13] Enoki T, Takai K (2008). Unconventional electronic and magnetic functions of nanographene-based host-guest systems. Dalton Trans.

[14] Kiguchi M, Takai K, Joly JVL, Enoki T, Sumii R, Amemiya K (2011). Observation of magnetic edge state and dangling bond state on nanographene in activated carbon fibers. Phys Rev B Cond Matter.

[15] Enoki T (2012). Role of edges in the electronic and magnetic structures of nanographene. Physica Scr.

[16] Rao CNR, Sood AK, Enoki T (2012). Magnetism of nanographene. Graphene: Synthesis, Properties, and Phenomena. Chapter 4..

[17] Enoki T, Ando T (2014). Physics and chemistry of graphene: graphene to nanographene.

[18] Murakami M (2006). Dimensionality and applications of various graphite carbons. Rev High Press Sci Technol.

[19] Sattler K (2010). Handbook of nanophysics.

[20] Mansour AE, Dey S, Amassian A, Tanielian MH (2015). Bromination of graphene: a new route to making high performance transparent conducting electrodes with low optical losses. ACS Appl Mater Interfaces.

[21] Kuila T, Bose S, Mishra AK, Khanra P, Kim NH, Lee JH (2012). Chemical functionalization of graphene and its applications. Prog Mater Sci.

[22] Englert JM, Dotzer C, Yang G (2011). Covalent bulk functionalization of graphene. Nature Chem.

[23] Georgakilas V, Otyepka M, Bourlinos AB (2012). Functionalization of graphene: covalent and non-covalent approaches, derivatives and applications. Chem Rev.

[24] Friedrich JF, Wettmarshausen S, Hanelt S, Mach R, Mix R, Zeynalov EB, Meyer-Plath A (2010). Plasma-chemical bromination of graphitic materials and its use for subsequent functionalization and grafting of organic molecules. Carbon.

[25] Diyuk VE, Zaderko AN, Veselovska KI, Lisnyak VV (2015). Functionalization of surface of carbon materials with bromine vapors at mediate high temperature: a thermogravimetric study. J Thermal Anal Calorim.

[26] Gryn SV, Tsyrina VV, Kovalenko AS, Alekseev SA, Lisnyak VV, Ilyin VG (2009). Template-directed synthesis of dually porous periodic organosilicas with 1,5-bis-(2’-ethyl)-xylene bridging groups. Mater Chem Phys.

[27] Diyuk VE, Mariychuk RT, Lisnyak VV (2016). Functionalization of activated carbon surface with sulfonated styrene as a facile route for solid acids preparation. Mater Chem Phys.

[28] Cavallo G, Metrangolo P, Milani R, Pilati T, Priimagi A, Resnati G, Terraneo G (2016). The halogen bond. Chem Rev.

[29] Zhang C-Z, Li T, Yuan Y, Gu C-Y, Niu M-X, Cao H (2016) Effect of bromine substituent on optical properties of aryl compounds. J Phys Org Chem. doi:10.1002/poc.3620.

[30] Zhang C-Z, Wang CY, Im C, Lu Y, Wang CS (2009). Significant effect of bromo substituents on nonlinear optical properties of polymer and chromophores. J Phys Chem B.

[31] Chu B (1974). Laser light scattering.

[32] Papirer E, Lacroix R, Donnett JB, Nanse G, Fioux P (1994). XPS study of the halogenation of carbon black – Part 1. Bromination. Carbon.

[33] Vidic RD, Siler DP (2001). Vapor-phase elemental mercury adsorption by activated carbon impregnated with chloride and chelating agents. Carbon.

[34] Sasmaz E, Kirchofer A, Jew AD, Saha A, Abram D, Jaramillo TF, Wilcox J (2012). Mercury chemistry on brominated activated carbon. Fuel.

[35] Zheng J, Liu H-T, Wu B, Di C-A, Guo Y-L, Wu T, Yu G, Liu Y-Q, Zhu D-B (2012). Production of graphite chloride and bromide using microwave sparks. Sci Rep.

[36] Hanelt S, Friedrich JF, Orts-Gil G, Meyer-Plath A (2012). Study of Lewis acid catalyzed chemical bromination and bromoalkylation of multi-walled carbon nanotubes. Carbon.

[37] Buchenko VV, Rodionova TV, Sutyagina AS, Goloborodko AA, Multian VV, Uklein AV, Gayvoronsky VYa (2016). Optical properties of thin nanosilicon films. Optic Mater.

[38] Gayvoronsky VYa, Popov AS, Brodyn MS, Uklein AV, Multian VV, Shul’zhenko OO (2015). The effect of sintering temperature on linear and nonlinear optical properties of YAG nanoceramics. Nanocomposites, Nanophotonics, Nanobiotechnology, and Applications, Vol. 156..

