# Supplementary material for: Surface Response of Brominated Carbon Media on Laser and Thermal Excitation: Optical and Thermal Analysis Study
Source: Nanoscale Res Lett. 2017 Feb 23;12:146. doi: 10.1186/s11671-017-1873-7 (PMC5323332; doi:10.1186/s11671-017-1873-7)
Supplement: Supplementary file 1 — Electronic Supplementary Material. (PDF 495 kb) [file 11671_2017_1873_MOESM1_ESM.pdf]

Electronic Supplementary Material

Surface response of brominated carbon media on laser and thermal excitation: optical and thermal analysis study

Volodymyr V. Multian[1], Fillip E. Kinzerskyi[1], Anna V. Vakaliuk[2], Liudmyla M. Grishchenko[2], Vitaliy E. Diyuk[2], Olga Yu. Boldyrieva[2], Vadim O. Kozhanov[2], Oleksandr V. Mischanchuk[3], Vladyslav V. Lisnyak[2], Volodymyr Ya. Gayvoronsky[1]

[1]Institute of Physics, the National Academy of Science of Ukraine, pr. Nauky, 46, 03028 Kyiv, Ukraine.

[2]Chemical Faculty, Taras Shevchenko National University of Kyiv, 62a, Volodymyrska Str., 01601 Kyiv, Ukraine

[3]O. O. Chuiko Institute of Surface Chemistry, the National Academy of Science of Ukraine, 17, General Naumov Str., 03164 Kyiv, Ukraine

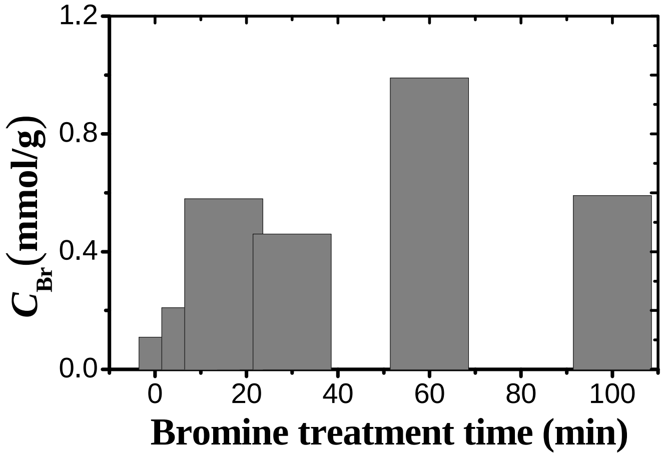

Fig. S1 Bromine concentration in the BrACFs against the treatment time

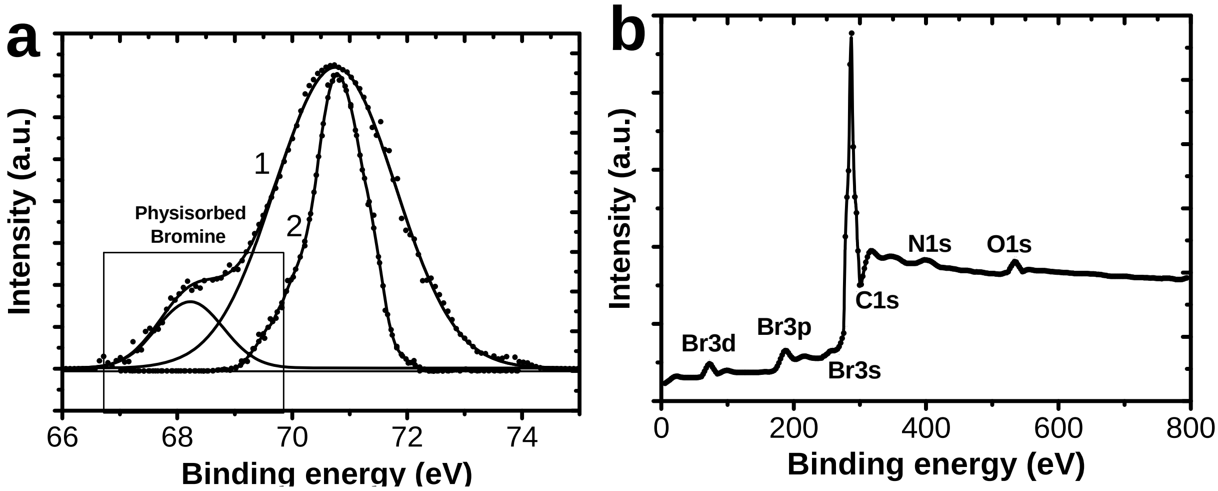

Fig. S2 XPS spectra **a** Br 3d core level for BrACF60 (1) and BrACF100 (2); **b** Typical wide spectrum; all are calibrated to the binding energy of C_{1s} photoelectrons at 284.6 eV

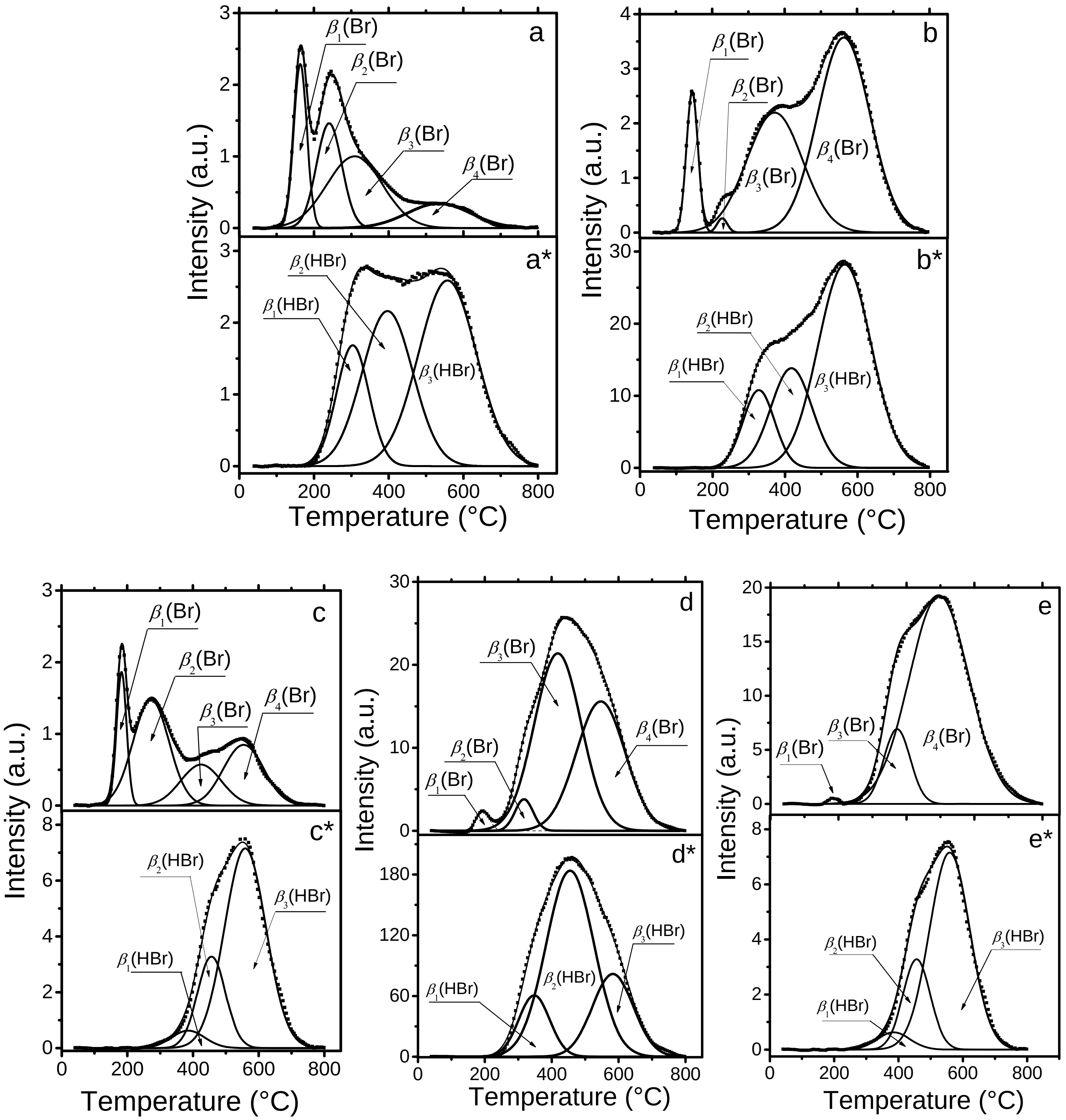

Fig S3. Temperature dependent mass selective m/z 79 – **a-e** and m/z 80 **a*-e*** positive ion current in TPD-MS analysis. **a** – BrACF5, **b** – BrACF10, **c** – BrACF30, **d** – BrACF60, **e** – BrACF100
